# Retraction: *Hippophae rhamnoides L.* (sea buckthorn) mediated green synthesis of copper nanoparticles and their application in anticancer activity

**DOI:** 10.3389/fmolb.2026.1809840

**Published:** 2026-02-19

**Authors:** Frontiers Editorial Office

The Journal retracts the 2023 article cited above.

Following publication, concerns were raised regarding the integrity of the images in the published figures. Figure 12 was previously published in:

Roy AM, Baliga MS and Katiyar SK (2005) Epigallocatechin‑3‑gallate induces apoptosis in estrogen receptor‑negative human breast carcinoma cells via modulation in protein expression of p53 and Bax and caspase‑3 activation. Mol. Cancer Ther. 4(1):81–90. doi: 10.1158/1535‑7163.81.4.1

The authors failed to provide a satisfactory explanation during the investigation, which was conducted in accordance with Frontiers’ policies. As a result, the data and conclusions of the article have been deemed unreliable and the article has been retracted.

This retraction was approved by the Chief Editors of Frontiers in Molecular Biosciences and the Chief Executive Editor of Frontiers. The authors received a communication regarding the retraction and had a chance to respond. This communication is recorded by the publisher.

